# Relationship between Humidity and Influenza A Viability in Droplets and Implications for Influenza’s Seasonality

**DOI:** 10.1371/journal.pone.0046789

**Published:** 2012-10-03

**Authors:** Wan Yang, Subbiah Elankumaran, Linsey C. Marr

**Affiliations:** 1 Department of Civil and Environmental Engineering, Virginia Tech, Blacksburg, Virginia, United States of America; 2 Department of Environmental Health Sciences, Mailman School of Public Health, Columbia University, New York, New York, United States of America; 3 Department of Biomedical Sciences and Pathobiology, Virginia-Maryland Regional College of Veterinary Medicine, Virginia Tech, Blacksburg, Virginia, United States of America; College of Medicine, Hallym University, Republic of Korea

## Abstract

Humidity has been associated with influenza’s seasonality, but the mechanisms underlying the relationship remain unclear. There is no consistent explanation for influenza’s transmission patterns that applies to both temperate and tropical regions. This study aimed to determine the relationship between ambient humidity and viability of the influenza A virus (IAV) during transmission between hosts and to explain the mechanisms underlying it. We measured the viability of IAV in droplets consisting of various model media, chosen to isolate effects of salts and proteins found in respiratory fluid, and in human mucus, at relative humidities (RH) ranging from 17% to 100%. In all media and mucus, viability was highest when RH was either close to 100% or below ∼50%. When RH decreased from 84% to 50%, the relationship between viability and RH depended on droplet composition: viability decreased in saline solutions, did not change significantly in solutions supplemented with proteins, and increased dramatically in mucus. Additionally, viral decay increased linearly with salt concentration in saline solutions but not when they were supplemented with proteins. There appear to be three regimes of IAV viability in droplets, defined by humidity: physiological conditions (∼100% RH) with high viability, concentrated conditions (50% to near 100% RH) with lower viability depending on the composition of media, and dry conditions (<50% RH) with high viability. This paradigm could help resolve conflicting findings in the literature on the relationship between IAV viability in aerosols and humidity, and results in human mucus could help explain influenza’s seasonality in different regions.

## Introduction

Influenza has distinct transmission patterns around the world. In temperate regions, influenza’s incidence peaks during the wintertime [1], [2], while in some tropical regions, the disease’s occurrence seems to coincide with the rainy season [3]–[5]. These patterns have triggered intense interest in unveiling the mechanisms behind them. However, a consistent explanation is lacking, despite nearly a century of investigation [6], [7].

Humidity has been identified as one factor that influences influenza’s seasonality [8], [9]. Previous studies have linked influenza’s high incidence in temperate regions to low humidity in wintertime [10]–[12]. This connection is further supported by laboratory studies indicating that influenza A virus (IAV) survives better at low relative humidity (RH) [8], [13]–[15].

Nevertheless, several important questions remain to be addressed. Firstly, the connection between high influenza incidence and low humidity fails to explain increased influenza activity in some tropical areas during the rainy season when humidity is maximal. Secondly, although laboratory studies consistently showed a high survival rate for IAV at low RH (<50%), results were discordant at medium to high (∼50% to ∼90%) RH [7]. Of the four studies cited most often, Hemmes et al. [8] and Harper [13] (referred to as H&H hereafter) found higher inactivation rates at both medium and high RH, while Shechmeister [15] and Schaffer et al. [14] (referred to as S&S hereafter) reported the highest inactivation rates at medium RH and moderate ones at high RH. The relationship between the viability of IAV in airborne droplets and ambient RH remains poorly defined and poorly understood.

Another unanswered question surrounds the mechanism by which humidity might affect IAV in airborne respiratory droplets [9]. After release from the respiratory tract, where RH is ∼100%, a respiratory droplet shrinks by 40–50% in diameter at RH below 90% due to evaporation [16]–[18]. As a result, concentrations of solutes in the droplet increase by up to 15 times, and solutes such as salts (e.g., sodium chloride (NaCl)) that are harmless at physiological levels may become harmful to the virus. For example, avian IAV has been reported to be less stable at salinities greater than 25 g L^−1^ [19]. Evaporation induces changes to IAV’s microenvironment inside droplets that may affect the virus’ viability, and the toxic effect of solutes may be enhanced at lower RH due to higher concentrations that result from greater loss of water. However, respiratory droplets also contain a variety of proteins [20], [21], and their interactions with salts at different RHs may complicate this picture under natural conditions.

We hypothesize that humidity mediates the survival of IAV in a droplet by controlling the extent of evaporation and thus solute concentrations in the droplet and that solute concentrations in the droplet define the relationship between RH and IAV viability. We designed a simple experiment to test the effects of salts and proteins on the viability of IAV and, for the first time, to determine its relationship with RH in human mucus. Our results resolve the aforementioned discrepancy in the literature. Based on these results, we propose a mechanistic explanation for the dependence of IAV’s survival on humidity and influenza’s transmission patterns in both temperate and tropical regions.

## Materials and Methods

### Ethics Statement

Collection and use of human mucus was approved by the Institutional Review Board at Virginia Tech. The ethics committee did not require consent to be obtained for this protocol.

### Cells and Virus

Madin-Darby canine kidney (MDCK) cells were maintained in Dulbecco's modified Eagle medium (DMEM) supplemented with 5% fetal calf serum (FCS). The stock of the IAV A/PR/8/34 (H1N1) was prepared with a plaque purified strain [22] in 10-day-old specific pathogen-free embryonated hens’ eggs. Virus stocks were stored as aliquots of allantoic fluid from infected embryos at −80°C until use. The titer of the virus stock was determined to be 1.78×10^8^ median tissue culture infective dose (TCID_50_) mL^−1^. All virus titration experiments were performed in MDCK cells.

### Mucus Specimen

A mucus specimen was collected from an infant 1 month of age in March 2011. The mucus specimen was allowed to desiccate in a vial and was reconstructed by dissolution in MilliQ water (5% w/v). To preserve the mucus structure, we first attempted to sterilize the mucus specimen by passing it through a 0.45-µm pore size filter. The specimen was not filterable, indicating its large molecular size. Thus, the reconstructed mucus was sterilized by heating at 65°C for 30 min and was checked for potential IAV contamination by TCID_50_ assay. No IAV was detected, and the specimen was stored at 4°C until use.

### Control of RH in a Desiccator

Two Petri dishes filled with 20 mL of salt solution (either potassium acetate, potassium carbonate, cobalt (II) chloride, or potassium chloride) or double distilled water were placed in a desiccator [23], [24]. Air circulation inside the desiccator was enhanced by a fan to accelerate the establishment of equilibrium RH between the salt solution and the air inside the desiccator. Before each experiment began, the desiccator was conditioned to the desired RH. The desiccator had to be opened briefly to insert the samples, and the system reestablished equilibrium within ∼10 min. The actual RH and temperature inside the desiccator were recorded at 1-min intervals during sampling with a temperature/humidity logger (OM-73, Omega Engineering, Inc., USA). RHs tested in model media included 17.5±1.0%, 27.6±0.9%, 42.0±0.8%, 44.1±1.2%, 50.2±0.7%, 59.5±1.1%, 76.1±0.7%, 84.1±0.8%, and 98.9±0.4%. RHs tested in mucus included 26.7±0.8%, 40.3±1.1%, 48.0±1.2%, 52.0±0.9%, 60.8±1.1%, 72.2±1.2%, 83.9±0.4%, and 99.1±0.4%.

### Exposure of IAV in Droplets to Various RH

We exposed IAV in droplets to specific RHs in the range of 17% to ∼100% at room temperature (20–24°C). The droplets consisted of four different types of media (Table 1), chosen to isolate the effects of salts and/or proteins on viability, or mucus. PBS and DMEM were used as surrogates of media that contain inorganic salts at physiological levels but no or negligible amounts of proteins. The third and fourth types of media were PBS and DMEM supplemented with 5% FCS, a source of proteins.

**Table 1:** Media used in studies of influenza A virus viability versus RH.

Study	Media	Salt content	Protein content	Other components	Reference
Hemmes *et al.* 1960	1 part allantoic fluid and one part2% Difco peptone	∼2.3 g L^−1^ (0.4 g L^−1^ K^+^, 0.9 g L^−1^ Na^+^,0.9 g L^−1^ Cl^−^)^a^	10 g L^−1^ peptone	-b	[8], [43]
Harper 1961	Allantoic fluid diluted 1∶8 or 1∶10 incasein McIlvaine’s buffer (pH 7.2)	∼2.2 g L^−1^ (mainly Na_2_HPO_4_) a	∼1.9 g L^−1^ mainly casein	-b	[13], [43], [44]
Shechmeister 1950	Allantoic fluid in 0.1 M Sorensen’sphosphate buffer (pH 7.1)	19.6 g L^−1^ (8.09 g L^−1^ NaH_2_PO_4_, 9.51 gL^−1^ Na_2_HPO_4_)	∼1 g L^−1 ^in allantoicfluid	-b	[15], [43]
Schaffer *et al.* 1976	MEM	9.88 g L^−1^ (6.8 g L^−1^ NaCl, 2.2 g L^−1^NaHCO_3_, and others)	0	Amino acids, vitamins,glucose, others	[14], [43], [45]
	MEM+0.1% BSA	9.88 g L^−1^	1 g L^−1^	Same as above	
	Allantoic fluid	∼4.7 g L^−1^ (0.8 g L^−1^ K^+^, 1.8 g L^−1^ Na^+^,1.8 g L^−1^ Cl^−^)^a^	∼1 g L^−1^		
This work	PBS (pH 7.2)	9.55 g L^−1^ (8 g L^−1^ NaCl, 0.2 g L^−1^ KCl,1.15 g L^−1^ Na_2_HPO_4_, 0.2 g L^−1^ KH_2_PO_4_)	0	None	
	PBS+5% fetal calf serum (FCS)	Same as above	∼3.5 g L^−1^	Other componentsfrom FCS	
	Dulbecco's modified Eaglemedium (DMEM)	10.92 g L^−1^ (6.4 g L^−1^ NaCl, 3.7 g L^−1^NaHCO_3_)	0.42 g L^−1^	Amino acids, vitamins,glucose, others	
	DMEM+5%FCS	Same as above	∼3.9 g L^−1^	Same as above	

a Estimated;

b Detailed composition of allantoic fluid is unknown.

Ten µL of stock PR/8 IAV was added into 90 µL of either PBS, PBS+5% FCS, DMEM, DMEM+5% FCS, or mucus to produce spiking solutions. The spiking solutions were distributed onto a 12-well cell culture plate, 1 µL per droplet, 10 droplets per well, and 3 replicates (i.e., 3 wells) for each medium. The plate was immediately placed into the desiccator and was incubated at a specific RH at room temperature for 3 h for model media and 2 h for mucus. At the end of the period, the virus in each well was collected with 1 mL of DMEM supplemented with 1 µg mL^−1^ TPCK trypsin (collection medium), by pipetting the medium ∼10 times to rinse the virus from the well. The spiking solutions were stored on ice during the incubation period, and 10 µL of each spiking solution was supplemented with 990 µL of collection medium for use as a control. Samples and the corresponding controls were titrated at the same time by TCID_50_ assay either immediately after collection or were stored at −80°C until testing.

### Observation of Transformation of Droplets under Varying RH Levels

Droplets of PBS, PBS+5% FCS, DMEM, or DMEM+5% FCS (all without addition of IAV) in 1-µL volumes were distributed on a 12-well cell culture plate and incubated under different RH levels as described above. Plates were taken from the incubator and observed immediately under an inverse light microscope (100X). Photos were taken from a camera connected to the microscope. To determine the time needed for a droplet of 1 µL to dry out, droplets of PBS, PBS+5% FCS, DMEM, or DMEM+5% FCS were distributed on a glass slide, left under the microscope and observed continuously until the droplet crystallized. Ambient RH was 49.4±0.2% in the microscope room.

### Calculation of Viral Decay

Percentage viability was calculated as
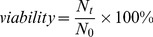
(1)where *N_t_* and *N_0_* are, respectively, the final and initial titers of virus. The log decay rate was calculated as
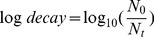
(2)
*Nt* was set to 1 for negative samples to avoid dividing by 0.

### Estimation of Solute Concentration in Equilibrium

The molality of NaCl in the resulting droplet at a specific RH was calculated using Microsoft Excel’s solver tool according to an empirical relationship [25]:
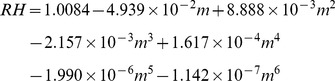
(3)where *m* is the molality of NaCl (mol (kg water)^−1^). The concentration of NaCl was then calculated based on *m*:
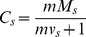
(4)where *Cs* is the concentration of solute in equilibrium; *Ms* is the molecular weight of NaCl (58.44 g mol−1); vs is the molar volume of NaCl (2.7×10−2 L mol−1); and the number 1 in the denominator represents the volume of 1 kg of water, i.e., 1 L.

## Results

### IAV Viability in Model Media v. RH

**Figure 1:**
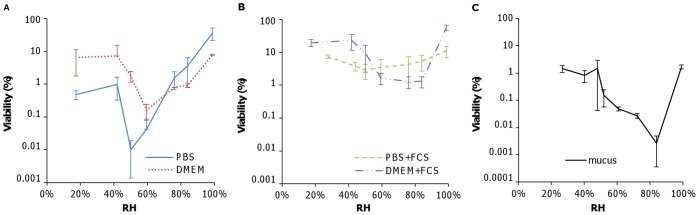
Relationship between RH and IAV viability in (A) media with mainly salts, (B) media with salts plus proteins, and (C) mucus. Error bars denote standard deviations.

As shown in Figure 1A and 1B, IAV viabilities in four model media containing various combinations of salts and proteins were highest at extremely high (∼100%) and low (<50%) RH and minimal at medium (50 to 84%) RH. At ∼100% RH, viabilities ranged from 8% to 57%, namely, 36±15% in PBS, 7.8±0.27% in DMEM, 11±4.1% in PBS+FCS, and 57±11% in DMEM+FCS of input virus. At ∼84% RH, viabilities were much lower (∼1–5%) but were of the same order of magnitude in all four media.

At RH between 50% and 84%, the trends in viability diverged dramatically, depending on whether the media contained proteins or not. The viability in PBS decreased with decreasing RH and reached a minimum at 50% RH. In DMEM, the trend was similar, but the minimum occurred at an RH of 60% (Figure 1A). In contrast, in PBS+FCS, viabilities did not change significantly over this range of RH, and in DMEM+FCS, viabilities increased slightly with decreasing RH (Figure 1B).

At RH below 50%, viabilities were higher in all types of media compared to those at medium RH, although viability in PBS alone was significantly lower than in the three other media, possibly due to the lack of proteins.

We repeated the experiment to confirm these results. The percent viabilities recovered varied slightly, but trends in viability versus RH were similar.

### IAV Viability in Mucus v. RH

We tested the changes in viability of IAV spiked into human mucus at RH levels ranging from ∼26 to ∼100%. As shown in Figure 1C, at ∼100% RH, the viability of IAV was only 1.7±0.26%, an order of magnitude lower than in model media. The finding that viability was highest at this extremely high RH was consistent with results in other media. At RH between ∼50% and 84%, viabilities increased dramatically with decreasing RH. Only 0.0027±0.0023% was recovered at 84% RH, compared to 1.5±1.4% recovered at 48% RH, a decrease of over two orders of magnitude. At RH below 48%, viabilities leveled off and remained around 1%. We repeated the experiment at four (27%, 60%, 84%, and 99%) of the six RHs, and the resulting viabilities followed the same trend as shown in Figure 1C. With spiking titers of 5.1±2.6×10^4^ TCID_50_, no viable IAV was detected in 5 out of 9 samples at ∼84% RH.

### Relationship between Viral Decay and Salt Concentrations

**Figure 2:**
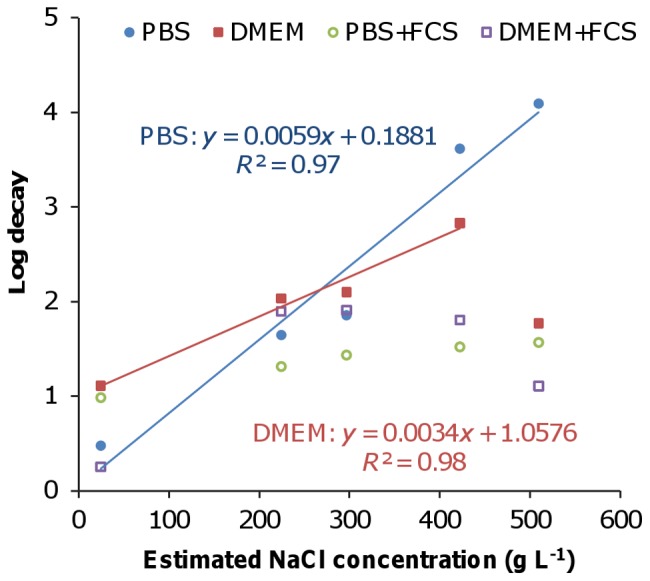
Viral decay over 3 h versus NaCl concentration in droplets consisting of four types of media.

The relationship between viral decay rates and salt concentrations in the droplets is shown in Figure 2. The four media used in this study and the mucus specimen contained multiple types of salts (Table 1). However, as NaCl was the major salt in all media and the mucus, we estimated the total concentrations of salts in the droplets at equilibrium in terms of equivalent amounts of NaCl; concentrations were estimated empirically [25]. The concentration of NaCl in a droplet is related to RH; however, as indicated by the polynomial function in Eq. 3, the correlation is not simply linear. The viral decay rate in PBS droplets increases linearly with NaCl concentration in the range of 25–510 g L^−1^, corresponding to 99–50% RH, respectively (*R^2^* = 0.97). Concentrations are lower at high RH because there is less evaporation and vice versa. Likewise, the viral decay rate in DMEM droplets also increases linearly with NaCl concentration up to 420 g L^−1^, corresponding to 60% RH (*R^2^* = 0.98). In contrast, in media containing FCS, viral decay rates remain constant regardless of NaCl concentration.

### Efflorescence

At RH below ∼50%, the relationship between IAV decay rates and NaCl concentrations is no longer valid. NaCl reaches its solubility in a droplet (310 g L^−1^) at 75% RH, and evaporation produces a supersaturated solution at RH<75%. The NaCl concentration can rise as high as 580 g L^−1^ before the droplet crystallizes [25], [26]. The critical RH at which a droplet crystallizes is termed the efflorescence RH (ERH), and it depends on the composition and size of the droplet [27].

**Figure 3:**
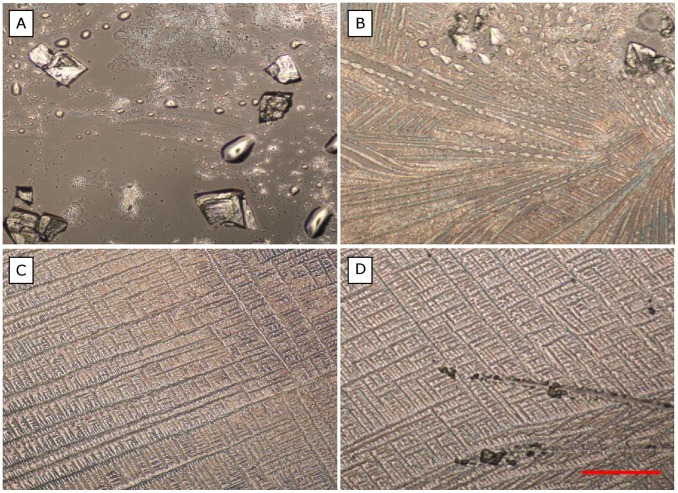
Crystals of the four media: (A) PBS, (B) PBS+FCS, (C) DMEM, (D) DMEM+FCS. Light microscope, 100X magnified; scale bar = 20 µm.

We observed the droplets of each medium under a microscope immediately after incubation at different RHs and found that no crystals formed at RH>60%, while droplets of all media crystallized at an RH just below 50% (Figure 3). Crystallization occurred within ∼10 min. Crystallization in DMEM, possibly at a lower NaCl concentration (corresponding to higher RH) than in PBS, due to a different ERH of the media, could explain the lower decay rate in DMEM at a concentration of 510 g L^−1^ (50% RH). Knowing the ERH of each media would lend further support to this idea, but the ERH of a solution is difficult to calculate and can only be measured accurately through specialized laboratory experiments [27].

## Discussion

### Hypothesis to Explain the Relationship between RH and Viability in Laboratory Studies

**Figure 4:**
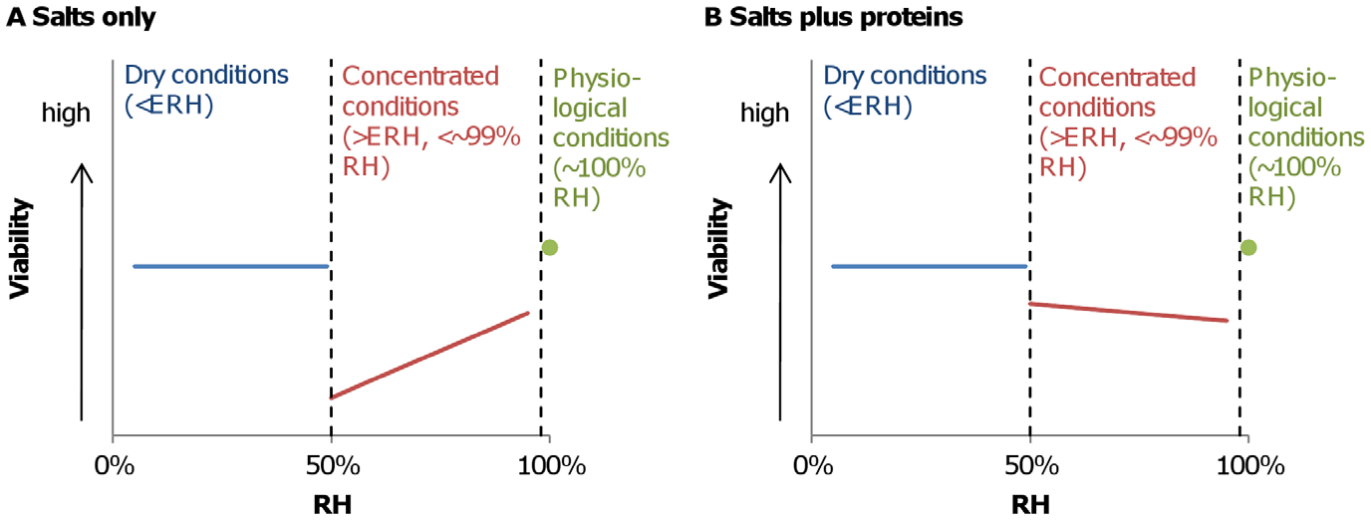
Hypothesized relationship between RH and IAV viability in (A) droplets containing salts only and (B) droplets containing salts plus proteins.

We postulate that there exist three regimes governing the viability of IAV in droplets, defined by ambient RH and shown in Figure 4: (1) physiological conditions (∼99 to 100% RH), where solute concentrations remain at levels harmless to IAV and viability is maintained, (2) concentrated conditions (∼50 to ∼99% RH), where evaporation leads to elevated salt concentrations that may be harmful to the virus, and (3) dry conditions (<50% RH), where solutes crystallize, all water is lost, and IAV viability is maintained.

In a saline droplet, the elevated salt concentrations that result from evaporation are likely to be toxic to the virus, but such deleterious effects would be eliminated when the solution crystallizes. Therefore, the minimum viability would be expected at an RH just above the ERH of the salts contained in the droplet, when water is still present and solute concentrations are maximal. The relationship between RH and IAV viability in a saline droplet would thus be similar to that shown in Figure 4A. However, the presence of proteins in the droplet may alter this relationship. It is possible that the interaction between proteins, salts, and the virus mitigates the adverse effects of salts under concentrated conditions. Therefore, the virus would maintain high viability under physiological and dry conditions and moderate viability under concentrated conditions in a droplet composed of both salts and proteins (Figure 4B). In fact, viability may increase with decreasing RH, as we found in DMEM+FCS and mucus, possibly due to protection provided by proteins at elevated concentrations [28], [29].

A study on Langat virus supports our hypothesis. Benbough [30] reported similar V-shaped curves of viability versus RH for Langat virus in aerosols composed of salt solutions. Of four RHs tested (i.e., ∼25%, 50%, 70%, and 95%), the minimum viabilities were ∼1% in NaCl and ∼10% in KCl, both at ∼50% RH, and zero in LiCl at RH<50%. Thus, the minimum viability of Langat virus in aerosols composed of an NaCl solution occurred at an RH close to the ERH of NaCl (i.e., 43±3%) [27]. KCl has an ERH of 59% [27], and this RH was not tested, so it is unknown whether the virus’ viability would have been lower at this RH. The ERH of LiCl, if it exists, is outside the range of RHs tested; its deliquescence RH is ∼11% [24].

### Explanation for Discordant Findings in Literature

When reviewing the four aforementioned studies (H&H versus S&S), we noticed that the media used to produce airborne droplets containing IAV differed. All the media contained some salts; however, those in H&H contained substantially more proteins than did those in S&S (Table 1). We thus hypothesize that the conflicting results between S&S and H&H were due to the varying protein content of the media used in these studies. Our results with model media confirm this hypothesis: the trends in viability v. RH in media with mainly salts (S&S and this study) resemble the illustration in Figure 4A, while those in media with both salts and proteins (H&H and this study) resemble Figure 4B.

We showed that viral decay is correlated with the concentration of salts in saline droplets, which is controlled by RH through evaporation (Figure 2). Previous studies based on experiments in bulk salt solutions at concentrations up to saturation failed to detect such a correlation [31]. The supersaturated conditions to which the virus is exposed in aerosols cannot be achieved in bulk media, and so the phenomenon observed here can only be demonstrated through methods that enable supersaturation. The combination of highly elevated salt concentrations at medium RH and salt crystallization at the ERH may explain the trends observed in the two media containing mainly salts (i.e., PBS and DMEM) and in S&S. The RH corresponding to the maximal decay rate may differ between media, possibly due to different ERHs for media of different compositions [27]. The minimum viabilities occurred between 40–70% RH, mostly around 50% in Schaffer *et al.* [14] and 58–60% in Shechmeister. [15] For comparison, we found minimum viabilities at 50% in PBS and 60% in DMEM.

As did H&H, we found that proteins had a “protective” effect for IAV under concentrated conditions at medium RH, although the reasons for it are still unknown. One possibility is that proteins in aerosol droplets could become enriched around viruses due to mutual hydrophobicity and provide some protection against concentrated salts. Although this hypothesis is highly speculative, it is supported by the fact that mucin glycoproteins usually serve as a barricade against potential pathogens including viruses and bacteria by specific or non-specific binding [28], [29].

### Relationship in Mucus

Respiratory tract mucus is a complicated combination of water (∼95%), inorganic salts (∼1%), and various macromolecular organic compounds including glycoproteins (i.e., mucins) and lipids [20], [21], [32]. It has been shown to be protective for IAV at low RH [33], [34]. However, the relationship between viability of IAV in mucus and RH is still not completely clear.

We initially conducted experiments with mucin extracts from porcine stomach (Type II, Sigma-Aldrich). Results indicated that IAV infection was blocked, probably by the bound sialic acid on the mucin. Similarly, glycans on human mucus could prevent IAV attachment to the cell in the TCID_50_ assay. However, such an effect was not significant according to our experiment. The titer of IAV spiked in the human mucus specimen was 5.1±2.6×10^7^ TCID_50_ ml^−1^ after being kept on ice for 2 h, with a spiking titer of 1.78×10^8^ TCID_50_ ml^−1^. In addition, reporting results in mucus samples as the ratio of the recovered viability after incubation at a specific RH over the initial viability, both tested in mucus, should control for any potential blocking effect due to glycans on mucus. Therefore, results reported here reflect the effect of RH.

In accordance with the literature [33], [34], we found higher viabilities at low RHs (<50%). The finding that IAV survived best at ∼100% RH, a condition which has not been examined previously, helps complete the understanding of the response of the virus to varying RH. The relationship in mucus bears some similarity to that in media with salts plus proteins, particularly DMEM+FCS (i.e., higher viabilities at ∼100% RH or RH<50% and much lower ones at medium RH ranging from ∼50% to 84%). However, viability was much more sensitive to RH in mucus than in synthetic model media. A small change in RH from 48% to 52% reduced the viability 10-fold, and viability at ∼84% RH was >500 times lower than at 48% RH. For comparison, in model media, viabilities varied by only an order of magnitude over the same RH range, which is consistent with past studies [8], [13]. Thus, RH might have a greater effect on IAV’s survival in mucus than has been demonstrated in past studies with synthetic media. These results underscore the potential impact of RH on the virus’ survival in its natural aerosol carrier during transmission.

### Implications for Influenza’s Transmission Patterns

Many mechanisms have been proposed to explain influenza’s seasonality, including (1) environmental and climatic factors (e.g., temperature, relative or absolute humidity, and solar intensity), (2) host behavioral changes (e.g., school schedule and increased crowding during winter or rainy seasons), and (3) oscillations in host immunocompetence (e.g., vitamin D levels and melatonin levels) [7], [35]. A recent review by Tamerius *et al.* [7] assesses the feasibility of various mechanisms and concludes that the central questions in influenza seasonality remain unresolved. Our study was designed to focus on the effect of humidity, and we conducted all experiments at room temperature. In indoor environments, where infection is more likely to occur due to the much larger amount of time spent there and the greater spatial density of potential hosts, temperature tends to fall in a narrow range around 20°C. With this restriction, we were not able to distinguish between the effects of relative v. absolute humidity.

Our findings in human mucus could help explain, at least in part, the transmission patterns of influenza. In temperate regions, wintertime heating reduces RH in the indoor environment to low levels, usually <40% [36], [37]. Low RHs not only help preserve the viability of IAV but also enable IAV carrier aerosols to persist longer in air because of their smaller size and lower settling velocities that result from more vigorous evaporation [16]. Thus, transmission of influenza in temperate regions could be enhanced in winter primarily via the aerosol route. In tropical regions, high temperatures may suppress transmission, particularly through the aerosol route [38], [39]. However, lower temperatures and near-saturated RH during the rainy season create an opportunity for transmission via different mechanisms for large droplets v. very small aerosols. Large droplets would settle more quickly due to gravitation because they do not shrink as much at ∼100% RH (only to 93% of their original diameters at 99% RH, and 76% at 98% RH [16]). Once settled on a surface, they may serve as a reservoir for contract transmission [39], [40] since IAV is well preserved at ∼100% RH, as shown in this study. On the other hand, submicron aerosols such as those exhaled in human breath [41] would remain aloft, and thanks to the lower temperatures and suitable RHs for survival, transmission by these submicron droplets via the aerosol route might still be effective.

### Limitation and Directions for Future Study

In this study, droplets of 1 µL, rather than smaller aerosols [42], were used to simulate the interplay of humidity, droplet evaporation, solute concentrations in the droplet, and virus viability. It takes ∼10 min for such droplets to dry out completely at ∼50% RH, considerably longer than for much smaller droplets (e.g., <1 s for a respiratory droplet 20 µm in diameter [17]). The legitimacy of extrapolating our results to aerosols expelled from human respiratory tract thus depends on whether the dynamics of evaporation are critical for IAV decay. Our results in model media are comparable to those conducted in aerosols [8], [13]. Harper [13] recovered 66–126% of IAV in aerosols 1 s after spraying, when evaporation was completed. The high recoveries indicate that the effect of the evaporation process itself on viral decay is negligible. Even if it were critical, such an effect was controlled for when comparing viral decay in the same type of medium versus RH, since all the droplets experienced a similar rate of evaporation. Nevertheless, verifying these results in aerosols is warranted.

This study proposes a new mechanistic basis for the effect of RH on IAV viability in droplets. However, it does not address the mechanism by which salts affect IAV viability at a molecular level, nor does it explain how the presence of proteins alters the relationship. In addition, the relationship in mucus differed slightly from that observed in model media. Given the complex composition and unique nature of mucus, mechanisms governing the relationship in mucus might differ from those in model media.

### Conclusions

This study reports novel data on the response of IAV in droplets of model media to varying RH, including extreme conditions that have never been studied, and for the first time presents the relationship between IAV viability in human mucus and humidity over a large range of RH. Results suggest that there exist three regimes of IAV viability defined by RH. We provide a mechanistic explanation for these regimes, based on droplet evaporation, a subsequent increase in solute concentrations in the droplet, and their effect on the virus. Our theory also explains the conflicting findings in the literature about IAV viability in airborne droplets [8], [13]–[15]. We further outline a new perspective on the dependence of IAV’s transmission on humidity, which introduces a possible explanation for influenza’s seasonal patterns in different regions.
